# Exploring the Morphospace of Communication Efficiency in Complex Networks

**DOI:** 10.1371/journal.pone.0058070

**Published:** 2013-03-07

**Authors:** Joaquín Goñi, Andrea Avena-Koenigsberger, Nieves Velez de Mendizabal, Martijn P. van den Heuvel, Richard F. Betzel, Olaf Sporns

**Affiliations:** 1 Department of Psychological and Brain Sciences, Indiana University, Bloomington, Indiana, United States of America; 2 Department of Medicine, Indiana University, Indianapolis, Indiana, United States of America; 3 Indiana Clinical and Translational Sciences Institute, Indianapolis, Indiana, United States of America; 4 Department of Psychiatry, University Medical Center Utrecht and Rudolf Magnus Institute of Neuroscience, Utrecht, The Netherlands; INSERM & Universite Pierre et Marie Curie, France

## Abstract

Graph theoretical analysis has played a key role in characterizing global features of the topology of complex networks, describing diverse systems such as protein interactions, food webs, social relations and brain connectivity. How system elements communicate with each other depends not only on the structure of the network, but also on the nature of the system's dynamics which are constrained by the amount of knowledge and resources available for communication processes. Complementing widely used measures that capture efficiency under the assumption that communication preferentially follows shortest paths across the network (“routing”), we define analytic measures directed at characterizing network communication when signals flow in a random walk process (“diffusion”). The two dimensions of routing and diffusion efficiency define a morphospace for complex networks, with different network topologies characterized by different combinations of efficiency measures and thus occupying different regions of this space. We explore the relation of network topologies and efficiency measures by examining canonical network models, by evolving networks using a multi-objective optimization strategy, and by investigating real-world network data sets. Within the efficiency morphospace, specific aspects of network topology that differentially favor efficient communication for routing and diffusion processes are identified. Charting regions of the morphospace that are occupied by canonical, evolved or real networks allows inferences about the limits of communication efficiency imposed by connectivity and dynamics, as well as the underlying selection pressures that have shaped network topology.

## Introduction

Characterizing the communication efficiency of a complex network should take into account dual sets of constraints, imposed by the topology and by the dynamical process operating on it. In this regard it is crucial to know whether communication is better described by *routing (navigation)* or by *diffusion* processes. These two types of dynamics differ radically in terms of whether interactions, matter and energy flows, or communication along paths occurs with partial or full knowledge of the global structure of the network [1], [2]. On the one hand, a routing/navigation process implies that communication flows from a specific source to a specific target along the fastest or most direct route, which implies global knowledge about the network topology. On the other hand, a diffusion process implies that communication occurs in the absence of specific targets, or that, even if targets are specified, a lack of knowledge about global network topology prevents particles or messages from taking shortest paths. To some extent, undirected diffusion or spreading occurs in social networks (innovations [3], rumors [4], contagious diseases [5]), and possibly also in neuronal communication [6].

One of the most widely used efficiency measures is defined as the average of the inverse of shortest path lengths between every pair of nodes in a graph [7], [8], here referred to as routing efficiency *E_rout_*. The use of *E_rout_* for characterizing network efficiency implies that particles or messages can selectively navigate through shortest paths and that shortening path lengths, for example through the addition of edges, automatically improves the routing efficiency of the system. However, when dynamics are best described by diffusion, shortening the path length, e.g. by adding edges, may decrease the efficiency of communication between pairs of nodes. Another difference between navigation and diffusion dynamics concerns the importance of resources, for example expressed by the number of particles needed to achieve reliable communication. In routing processes, even single particles or messages can reach their destinations along efficient paths, while in diffusion processes the ability to send multiple particles may greatly increase the efficiency of communication. Hence, characterizing the communication efficiency of a system requires considering a combination of three different characteristics: network topology (structure), the ability to find shortest paths among all alternatives (knowledge), and the capacity to increase message traffic (resources).

In this paper we first introduce and define a set of interrelated measures of communication efficiency for systems whose dynamics are based on diffusion processes. These measures capture the probability with which single particles travel through shortest paths, their average propagation velocity across the network, and the degree to which additional resources help the system to approach optimal performance. These measures of diffusion efficiency complement the classical measure of routing efficiency [8]. In the main part of the paper we take routing and diffusion efficiency as the principal axes for defining an “efficiency morphospace” [9] for complex networks, i.e. a space where each combination of routing and diffusion efficiency is associated with characteristic aspects of network topology. We explore this space following three different approaches: (i) by examining a number of canonical and idealized network models; (ii) by employing a multi-objective optimization strategy to evolve network topologies that either facilitate or impede routing and/or diffusion efficiency; and (iii) by investigating routing and diffusion efficiency in a collection of real-world systems, including brain, protein-interaction, genetic regulatory, social, virtual social, transportation and digital circuit networks. We frame our discussion in the context of what our exploration of a morphospace for communication efficiency in complex networks tells us about possible limits and selection pressures on efficient communication imposed by network structure, knowledge and resources.

## Materials and Methods

### Graph Theory

In this section we briefly define some basic graph theory concepts relevant to the work described in this article (see [7], [10] for detailed reviews on network theory). Let us define a binary undirected graph as 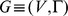, composed by a set of 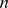 nodes 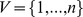 and a set of 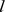 undirected edges 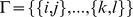, excluding self-connections. The graph's connection topology is described by a 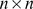 symmetrical adjacency matrix 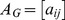, with 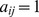 if edge 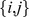 exists, and 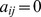 otherwise. The degree of a node 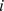, denoted by 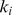 refers to the number of direct neighbors and can be expressed as 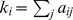. The average degree of a graph is the mean over all node degrees 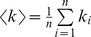 and the graph density is the fraction of edges that are present out of all possible, 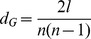. Assortativity is defined as the Pearson correlation coefficient of all degree-degree pairs of connected nodes [11]. The heterogeneity of the graph's degree distribution can be characterized by its Shannon entropy [12], given as 
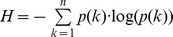
 as described in [13], [14]. The graph's community structure can be determined by identifying a partition into non-overlapping modules (using an optimization algorithm such as the Louvain method [15]) that maximizes a modularity metric [16]. All graph algorithms used in this article were implemented in Matlab and are available as part of the Brain Connectivity Toolbox (www.brain-connectivity-toolbox.net) [17].

### Markov Chain Theory

A Markov chain 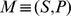 is formed by a set of states 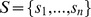, and a matrix of transition probabilities or *transition matrix* 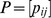 that characterizes the probability of going from one state 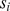 to another state 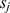 in one step [18]. A connected graph can be expressed as a Markov chain where states 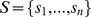 correspond element by element to the set of nodes 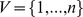.

Diffusion in networks is generally modeled as a random walk process, which in the simplest case involves the use of only local information about connectivity. In a binary network, the probability to go from one state (corresponding to the *source node*) 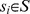 to another state (the *target node*) 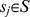 in one step is denoted by 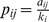 which assumes uniform probability of choosing one of the possible 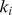 edges.

The next sections describe a set of measures characterizing communication efficiency. One measure has been introduced previously [8] and addresses communication along shortest paths, hence denoted here as routing efficiency. In addition, we introduce three related measures describing communication associated with diffusion processes, denoted shortest-path probability, diffusion efficiency and resource efficiency. All efficiency measures described in this study assume conservation of particles or walkers. The measures as defined here apply to connected undirected binary graphs, for which a diffusion process or random walk can be modeled by an ergodic (irreducible) Markov process [18]. Ergodicity is ensured by connectedness as it requires that any state can be reached from any other state in a finite number of steps. While most measures can be generalized to connected undirected weighted graphs, for simplicity we limit our analyses in the present paper to connected undirected binary graphs.

### Routing Efficiency

The shortest path length between two nodes of an undirected binary graph is defined as the minimum number of edges (and thus steps) that separate two nodes within a graph. The set of shortest path lengths between all node pairs is denoted by the symmetric matrix 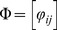. As defined in [8], assuming parallel communication across the network, a global measure of efficiency (originally termed global efficiency *E_global_* and here denoted as routing efficiency *E_rout_*), can be computed as
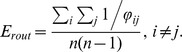
This measure assumes that communication operates in the shortest-path-regime.

### Shortest-Path Probability

Given a random walk process on a graph, an analytic expression can be derived that gives the probability that a single particle departing from a node 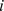 arrives at node 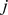 for the first time within exactly 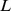 steps [19]. This criterion can be applied for each source-target pair by setting 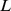 to their shortest-path-length. Let us denote by 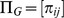 the 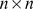 symmetric matrix containing, for each pair of nodes, the probability that a single particle going from node 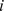 to node 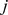 follows the shortest path (or one of them, if there exists more than one). Each entry 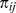 can be computed as
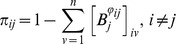
where matrix 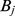 is the transition matrix 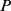 introduced above, but with all zeros in the 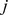-th column, i.e. with 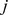 acting as an absorbing state [18], [19]. Evaluating shortest-path-lengths ensures that 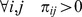. Hence, considering one particle, the average shortest-path probability of a graph is defined as
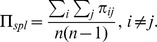



### Diffusion Efficiency

Let us denote by the matrix 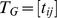 the mean first-passage time of an undirected connected graph 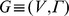 where nodes 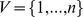 stand for states 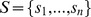 of the Markov chain. The Markov chain associated with an undirected connected graph ensures ergodicity. This property permits to compute the mean first-passage time [18] from the fundamental matrix 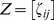 and the fixed row probability vector 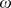 as
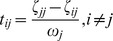
The probability vector 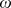 is the left eigenvector associated with the eigenvalue of 1, corresponding to the stationary solution of the underlying Markov process. The fundamental matrix 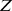 is computed as 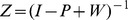 were 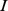 is an 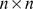 identity matrix, 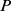 is the transition matrix defined above and 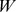 is an 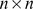 matrix with each column corresponding to the probability vector 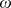 such that 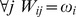. Analogously to *E_rout_*, we express by *E_diff_* the mean value of all inverse entries 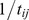 of the 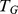 matrix excluding the main diagonal, yielding
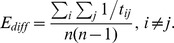



### Resource Efficiency

Let us define 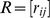 as the amount of resources (i.e. number of particles or messages) required to ensure with probability 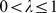 that at least one of them will, after starting at node 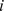, arrive at node 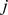 in exactly 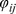 steps (i.e. as fast as possible given the graph structure):
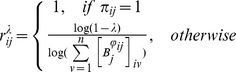
The simplest case arises when node 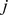 is the only neighbor of node 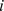 (i.e. 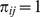) and thus just one particle is required for any value of 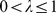.The term 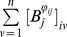 corresponds to 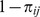, as explained above. Note that this equation differs from the one proposed in [19] in using the shortest-path-length of each pair instead of a fixed global number of steps for all pairs. Analogously to *E_rout_* and *E_diff_*, the efficiency of resources needed for a graph to operate in the shortest-path-regime with a fixed probability 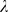 is defined as
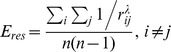
For the analyses performed in this paper we set 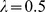.

### Scaling of Efficiency Measures

Graph measures are known to be strongly constrained by features such as network size or density [20]. Following an approach to scaling of network metrics applied to small-world attributes such as the clustering coefficient and shortest path length [21], we scale *E_rout_* of a given network relative to the average efficiency of one-dimensional lattices and random ensembles (denoted by 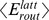 and 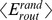 respectively) of the same size and density:
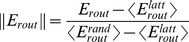
Analogously, scaled versions of 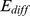 and 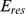, are obtained and denoted by 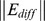 and 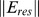 respectively.

### Efficiency Morphospace

The morphospace is a concept introduced originally in paleontology and evolutionary theory [9] to allow the systematic mapping of biological forms using structural parameters derived from a geometric model. These structural parameters define a space of all possible morphologies which specifies both existent and nonexistent forms. Here, the concept of morphology is extended to include network architectures, and the parameters defining the axes of the morphospace correspond to measures derived from network topology. Specifically, the scaled measures of routing and diffusion efficiency 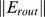 and 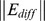 are taken to represent the principal axes of an “efficiency morphospace” within which networks are placed according to the level of communication efficiency they support. Exploring this morphospace allows probing the extent to which the space can be occupied by realizable network topologies, and what characteristic network features are encountered in different regions of the space.

### Canonical Network Models

Four canonical network models were used to capture the relationship between density, shortest-path probability and routing efficiency, selected because they capture relevant aspects of network organization encountered in a wide range of real-world systems. Models were Erdös-Rényi random graphs [22] (here implemented with a small variant to fix the overall density), networks generated by preferential attachment [23], one-dimensional lattices [24], and Watts-Strogatz small-world networks [24]. Details on how graphs corresponding to these canonical models were generated can be found in Text S1.

### Multi-Objective Optimization

The use of optimization algorithms operating on the efficiency morphospace allowed us to explore the commonalities and differences between 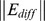 and 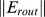 in connected undirected graphs, and the specific topological aspects that lead to such differences. The computational strategy we selected for optimization is an evolutionary algorithm operating on populations of graphs and performing incremental rewiring to change their topology. Population size, network size and density, as well as rewiring rate were selected to allow comprehensive exploration of the morphospace within the limits imposed by computational resources.

Simulations were carried out on populations (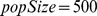) of undirected connected graphs of size 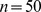 at two different densities determined by an average node degree 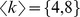. At the beginning of each experiment, 500 one-dimensional lattices were generated. In order to create some variance in the initial population while preserving connectedness, density and degree-sequence, 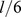 xswap rewiring steps were carried out on each lattice (see Text S1 for details of the randomization algorithm). Following this initialization, multi-objective optimizations [25], [26] were carried out, resulting in the formation of Pareto fronts [25] in the morphospace defined by our two principal efficiency measures, 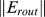 and 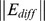. Four different types of optimizations were implemented and run independently. Each started from the same initial population, but employed a different fitness function to explore all four directions of the two-dimensional morphospace: maximizing 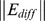 and minimizing 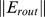 (front 1), maximizing both 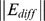 and 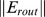 (front 2), minimizing 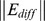 and maximizing 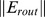 (front 3), and minimizing both 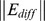 and 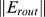 (front 4).

At each epoch, the Pareto front concept was applied to define survival criteria by partitioning the population into non-dominated and dominated solutions [25], which in our case are graphs. Hence a graph is said to be non-dominated (and thus a member of the Pareto front) if and only if no other graph exceeds it in the two gradients along 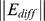 and 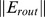 (the sign of each dimension is defined by the front and characterizes whether the objective is maximizing or minimizing each of the axes). Remaining graphs of the population not belonging to the front are said to be dominated. Pareto front members survive intact to the next population (next epoch). Finally, non-Pareto front members became extinct and were replaced in the next epoch by (randomly chosen) Pareto front members which were minimally rewired, with one edge added and one edge removed ensuring connectedness when preserving density and applying xswap when preserving degree sequence (see Text S1 for details). This process was continued over a maximum of 1000 epochs, unless the process was terminated earlier if one of two stopping criteria was reached. The first stopping criterion was based on the “age” of the Pareto front. If the age of Pareto front solutions exceeded, on average, 50 epochs, the optimization driving that front was stopped. The second stopping criterion was based on the balance between dominated and non-dominated graphs. If Pareto front members represented more than 95% of the total population, the evolutionary optimization driving that front was stopped. Two movies showing the advance of the four Pareto fronts, corresponding to the four fitness functions, for the two network densities 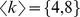 are available (Movie S1; Movie S2).

### Real-World Networks

A total of 23 real-world networks were analyzed (see Text S1 for details and Table S1 for network features). For all networks, the undirected un-weighted version of the giant component was used for all the analyses and for all network and efficiency measures.

## Results

### Communication Efficiency in Canonical and Idealized Network Models

**Figure 1:**
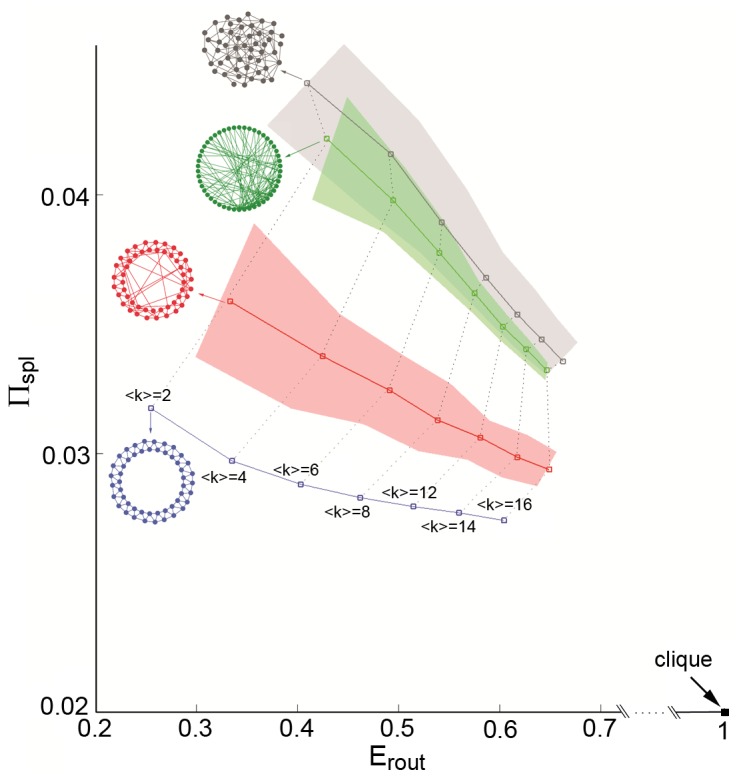
Relation of routing efficiency and shortest-path-probability for canonical network models. Scatter plot of 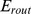 and 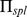 for four canonical network models at different densities: the Erdös-Rényi model (gray), the preferential attachment model (green), the Watts-Strogatz small-world model (red) and the one-dimensional lattice model (blue). The average degree is indicated at the plot line for the lattice model and dotted lines indicate equivalent average degrees for the rest of the models. Network size is fixed at 50 nodes. Each point represents mean values and shaded areas correspond to the 90% confidence interval. To obtain each data point (for each model and each average degree), 100 sample graphs were generated, except for the regular lattice given its deterministic nature. The clique coordinates are given as a reference point with maximum density and thus maximum 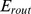 but very low 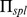. As the density increases, 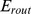 monotonically increases whereas 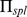 tends to monotonically decrease.

First we examined how increasing density affects 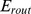 and 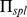 (Figure 1) in four canonical graph models: Erdös-Rényi random graphs, networks generated by preferential attachment, one-dimensional lattices, and Watts-Strogatz small-world networks. 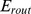, which represents the efficiency of the shortest-path-regime, increased in all models as the average degree of the network increased due to the addition of edges. In contrast, the probability 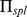 that a single particle travels along shortest paths in a diffusion process was found to be not only small (on the order of 10^−2^ for networks of 50 nodes) but also tended to decrease as network density increases. Over all network densities and at a given level of 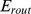, Erdös-Rényi random graphs afforded the highest 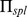 and one-dimensional lattice graphs afforded the lowest 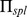.

**Figure 2:**
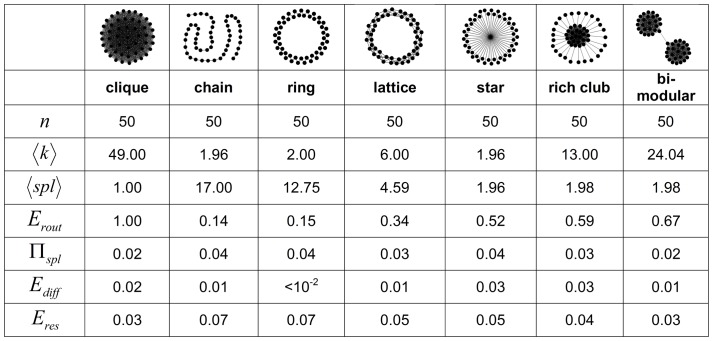
Graph and efficiency measures for seven idealized network topologies. Descriptors shown are: number of nodes 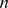, average degree 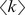, characteristic path length 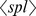, routing efficiency 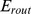, shortest-path probability 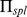, diffusion efficiency 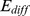 and resources efficiency 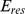.

Figure 2 summarizes non-scaled efficiency measures for various idealized network topologies, with 50 nodes and varying edge densities. A clique has the shortest possible path length and hence optimal 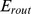, but also low 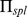 and 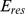, rendering this topology well suited for routing, but poorly configured for diffusion dynamics approaching the shortest-path-regime, either with a single or with multiple particles. In contrast, chain and ring topologies have long path length and low 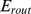, while also exhibiting high 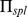, mainly due to the low network density which limits the total number of non-optimal paths and walks. 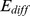 in chain and ring is low, indicating that a diffusion process on this graph is very inefficient. A regular lattice with 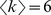 improves with respect to the ring (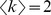) in both 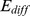 and 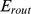. A star has high 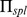, high 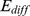 and moderately high 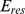 and 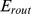, suggesting that the star topology is well suited for both routing and diffusion processes. Similar characteristics were found in a network containing a rich club [27]. Finally, a bi-modular network, while efficient for routing, is inefficient for diffusion since the presence of modules creates bottlenecks for inter-module communication, resulting in low 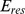 since many particles are needed to overcome these bottlenecks in inter-module communication. Larger networks (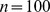 and 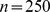) showed qualitatively similar results across these different network topologies.

### Evolving Networks in the Efficiency Morphospace

The characterization of these idealized network topologies provided some intuition about which network architectures promote different aspects of communication efficiency. However, the cases listed in Figure 2 differ greatly in density and degree sequence, both known to strongly affect virtually all graph measures. To more objectively characterize the relation of network topologies to measures of network efficiency, we implemented a multi-objective optimization strategy designed to search for network topologies in the two-dimensional efficiency morphospace formed by 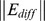 and 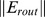. The algorithm allowed the connection pattern (including the degree sequence and the degree distribution) to evolve through a process of incremental rewiring while preserving connectedness and density (see Text S1 for details of the rewiring methods). Using a multi-objective fitness function, selection pressure was applied to drive networks in four different directions within the scaled efficiency space, resulting in four evolving populations whose leading fronts move towards high 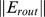 and low 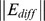 (front 1), high 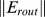 and high 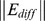 (front 2), low 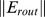 and high 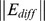 (front 3) and low 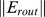 and low 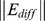 (front 4). Once evolutionary progress slowed, the fronts were considered to have reached an end state (Figure S1). At the end of each run, the surviving networks in each Pareto front can be said to jointly optimize the multiple objectives embodied in the fitness function.

**Figure 3:**
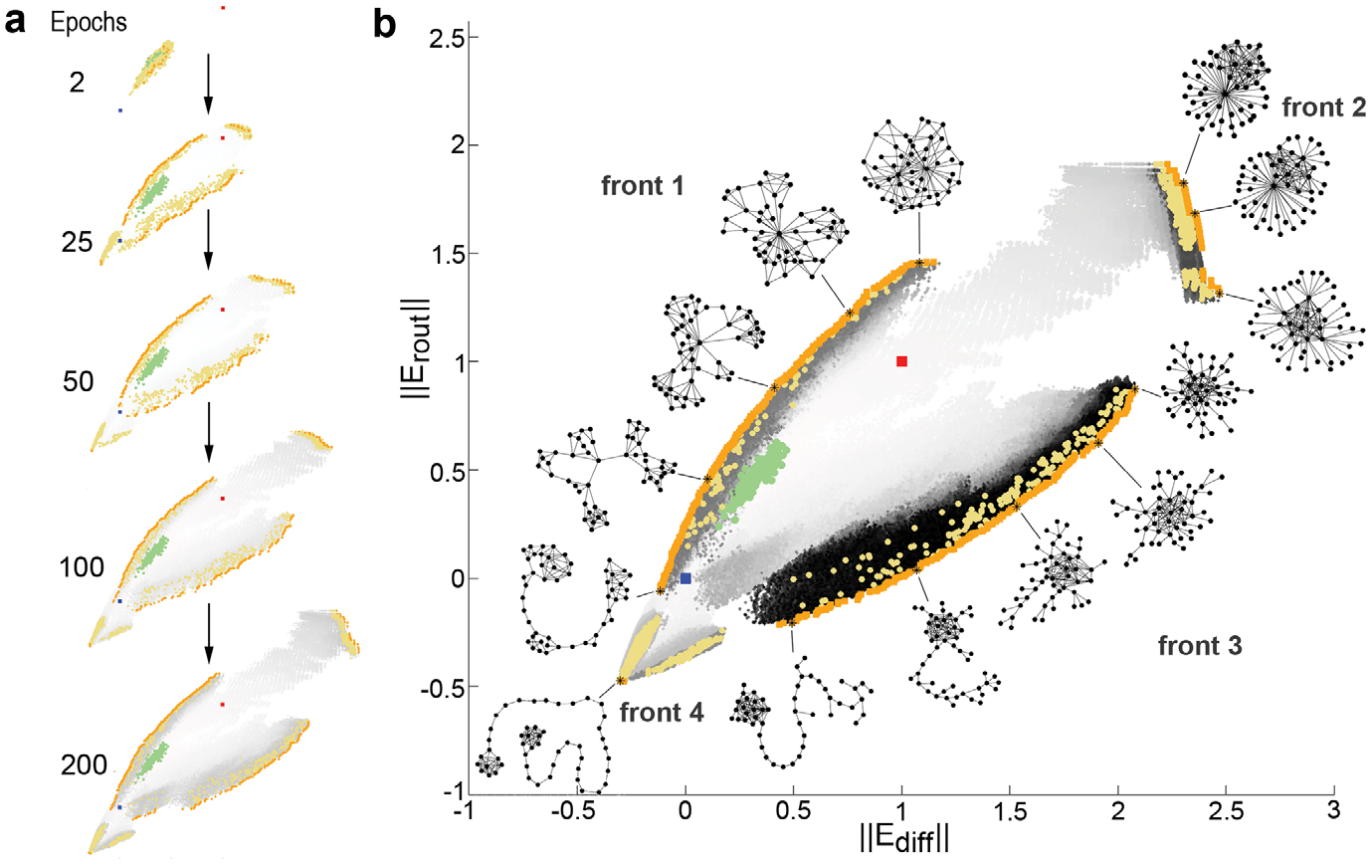
Multi-objective optimization in the efficiency morphospace. Results shown are for evolutionary processes driven by network efficiency measures for networks with 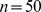 and 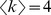. Blue and red squares indicate the reference points of regular lattices and randomized networks respectively. Green points indicate the initial seed population. Gray circles indicate evolving networks over epochs, with darker shades of gray indicating networks encountered in later epochs. Orange points show Pareto-front (non-dominated) solutions. (a) Snapshots illustrating the expansion of the Pareto fronts at epochs 2, 25, 50, 100, and 200. (b) Final solutions were reached after 517, 704, 977, and 433 epochs for fronts 1, 2, 3, and 4 respectively. Black asterisks denote positions of the example graphs shown in insets. Yellow points show dominated solutions of the final populations. Grey points show coordinates visited during the evolutionary process at different epochs (denoted by the gray-level).

Figure 3 shows a set of four evolutionary simulations, each employing one of the four fitness functions, for networks with 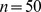 and 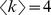 (for 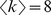 see Figure S2). Representative networks were selected across the entire length of each of the four final Pareto fronts corresponding to the evolved end states and were structurally characterized. Networks in front 2 were found to have evolved towards star-like topologies to the maximum extent permitted by the density imposed, while condensing all the remaining edges into a clique-like dense cluster. Networks in front 4 were found to have evolved towards chains as far as the imposed density permitted, with two dense clusters located at the extreme ends of the chain. Front 1 contained networks with topologies that were intermediate between those encountered in fronts 2 and 4. Along front 1, when moving away from the low-efficiency part of the front, networks gradually dissolved the chain topology into several linked clusters. Moving towards the high-efficiency part of front 1, star-like patterns began to emerge, resembling networks found in front 2. Front 3 exhibited a different transition from chain-like to star-like networks involving intermediate networks characterized by a set of densely interconnected high-degree nodes (akin to “rich club” organization [27]) that link to a number of peripheral low-degree branches. Figure S2 shows representative evolved network topologies for 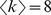, with all Pareto fronts showing very similar network characteristics to those seen for 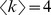.

**Figure 4:**
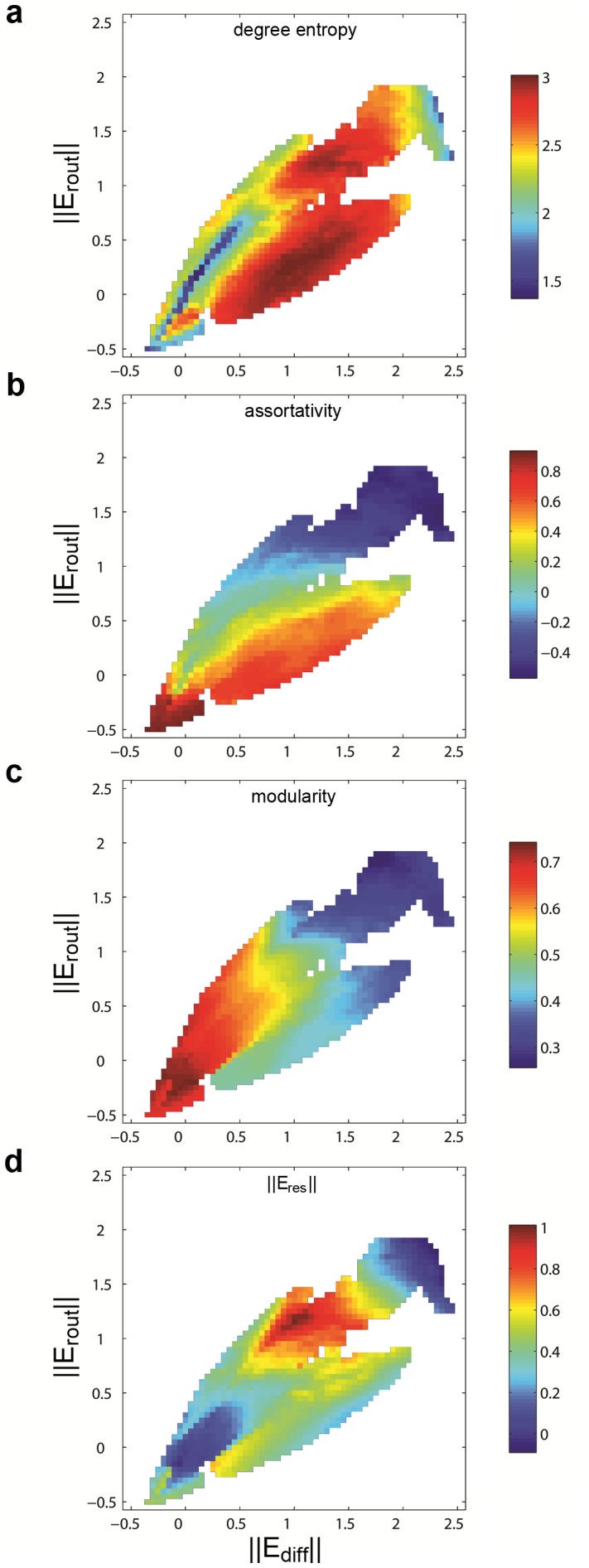
Graph measures for evolved networks. Results shown are for networks with 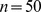 and 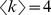. Heat maps are based on a square grid with cells measuring 0.05 units in each dimension. For each cell, graph measures coming from graphs falling on those coordinates at any epoch of the evolutionary processes (one for each front) were averaged. (a) Degree entropy. (b) Assortativity. (c) Modularity. (d) Scaled resource efficiency 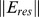.

Figure 4 shows graph metrics (the entropy of the degree distribution, assortativity, modularity and normalized resource efficiency) for network topologies encountered throughout the optimization process, mapped along the two dimensions of 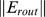 and 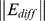 for 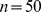 and 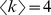 (for 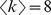 see Figure S3). Degree entropy was observed to be high on fronts 1 and 3, whereas it is lower on front 4, and lowest on front 2, due to the abundance of nodes with either low or high degree. Assortativity and modularity showed two main gradients across the space spanned by 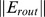 and 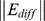. When moving from front 2 to front 4, networks proceeded from being highly disassortative to highly assortative, and from being moderately modular to highly modular. When moving from front 1 to front 3, networks proceeded from a mixture of non-assortative and disassortative networks, to assortative networks, and from mostly modular networks to less modular networks. Resource efficiency was highest in a region surrounding the random reference point, but lower on all the fronts. With respect to the measure of resource efficiency, lattice-like and star-like topologies behaved similarly when using multiple particles to overcome a lack of structural knowledge, but for different reasons. In lattices, almost any communication between two nodes required a number of particles to ensure using the shortest path with certain probability. In star-like topologies, fast communication between perimeter nodes and central nodes required one or only a few particles, while communication between any two perimeter nodes required a greater number of particles.

To further explore the role of the degree distribution in these evolutionary experiments, additional evolutionary optimizations were carried out where all rewiring steps were performed such that the initial uniform degree sequence (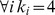) was maintained. Preserving the uniformity of the degree sequence was found to greatly restrict the range of 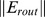 and 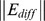 that can be reached by evolving networks (Figure S4), essentially limiting such networks to a narrow region spanning the lattice and random reference points with a very high positive co-variance between 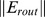 and 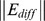. Thus, the uniformity of the degree sequence (which corresponds to zero degree-sequence entropy) had a strong impact on network communication by contracting the region of the morphospace that is accessible and at the same time creating a strong mutual linear dependency between routing and diffusion efficiency.

### Real-World Networks in the Efficiency Morphospace

**Figure 5:**
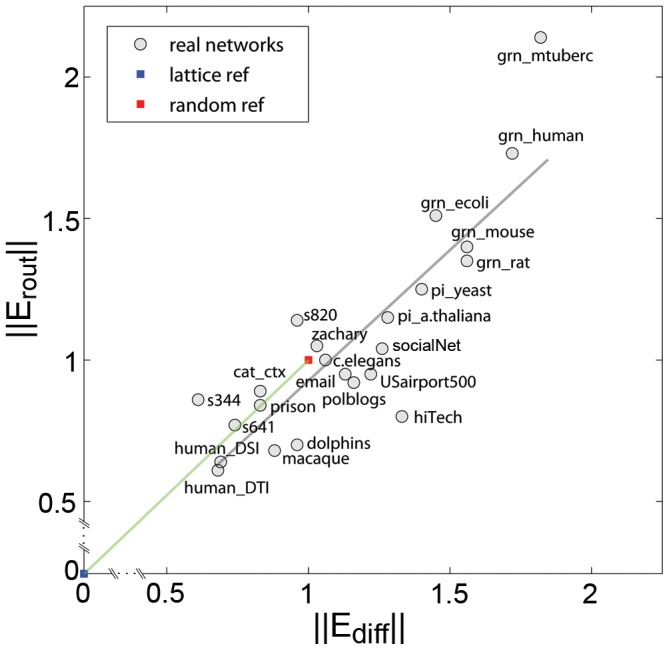
Placing real-world networks in the efficiency morphospace. The figure shows a scatter plot of 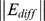 and 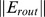 for 23 real-world networks (for description of data sets see Text S1). Blue and red squares indicate lattice and random reference points, respectively, linked by the green reference line. The gray line represents the linear regression across all 23 real-world networks (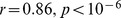).

Figure 5 shows the efficiency morphospace represented by 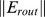 and 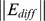 for 23 heterogeneous real-world systems, including brain, protein-interaction, genetic regulatory, social, virtual social, transportation and digital circuit networks (numerical values for network metrics are given in Table S1). Examining Figure 5, three basic observations can be made. First, it appears that most of the morphospace is empty, i.e. most combinations of 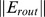 and 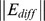 are not actually encountered in real-world networks. Second, networks found within the small region of the morphospace form an elongated cloud of points aligned with the positions of the two reference points for lattice and random topologies and their 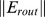 and 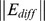 exhibits a strong and significant linear correlation (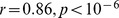). Third, most social, virtual social, digital circuit and transportation networks are located close to the random reference point. Protein interaction networks and genetic regulatory networks are the most efficient, exceeding the performance of equivalent random networks both in terms of diffusion and routing. Indeed, a visual inspection of these networks indicates the presence of numerous star-like patterns. Regarding brain networks, we observed a difference in networks recorded at macroscopic (whole-brain regional) and microscopic (single neuron) resolution. Whole-brain human brain networks acquired by diffusion imaging and tractography were placed within a lower efficiency regime compared to the single-neuron network of *C. elegans* which performed very similar to random networks for both efficiency measures.

## Discussion

The efficiency of communication in complex networks is of central interest across many disciplines studying physical, social, technological or biological systems. Here we explore the relation between different measures of communication efficiency based on routing or diffusion processes, and different aspects of network topology. This exploration is carried out within an efficiency morphospace whose two axes are defined by scaled measures of routing and diffusion efficiency. Complex networks are positioned in this space depending on the level of communication efficiency they support. We explore this space by adopting three different approaches. An examination of idealized topologies, evolved architectures and real-world networks reveals characteristic differences in the way different network architectures facilitate or impede communication via routing or diffusion.

Communication efficiency has been characterized in different ways, employing routing [28], diffusion [29], [30], or more complex navigation models that include, for instance, limited information [31], communication based on hierarchical structures [32], [33], [34], selection principles with a trade-off between routing and congestion [35], or walkers capable to benefit from learning [36]. Contrasting different models for communication processes such as routing and diffusion strongly suggests that when considering global measures of network efficiency it is important to recognize that operating in the shortest-path regime requires that system elements can access complete knowledge of the global network structure. This point is particularly salient for models of network communication that are based on diffusion dynamics, traditionally characterized by random walks of single particles across the network [1], [18]. Here system elements do not possess knowledge about the global network topology and instead draw exclusively on local information when moving towards one of the neighbors of the node currently visited. This limited knowledge makes it highly improbable that a diffusion process can proceed along the shortest paths between nodes. As a foundation for exploring the relation of communication efficiency to network topology we introduced a novel set of measures designed to capture the efficiency of communication based on diffusion processes, which complement the classic measure of routing efficiency [8]. Scaling both measures to appropriate random models allowed us to position different network topologies within the space defined by 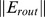
and 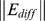 and draw relations between topology and efficiency.

Star-like topologies exhibit high 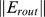 and high 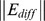, but their high communication efficiency comes at the expense of low 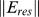 and high vulnerability to targeted attack on central nodes [37]. Modular networks exhibit higher 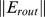 than 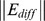, suggesting a trade-off between shortest paths that permit efficient routing, and high clustering that prevents diffusing messages or random walkers to leave local communities and reach other parts of the network, an attribute that also results in low 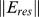. High 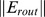 and low 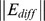 may be desirable in many real systems as the dispersal of noisy perturbations, rumors or contagious diseases remains limited to restricted parts of the network. The inverse pattern, low 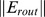 and high 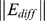, is encountered in networks with a central and highly connected ‘core’ or ‘rich club of hubs’ linked to low density branches, with improved robustness as indexed by high degree entropy [13]. Finally, long chain structures linking highly connected modules represent network topologies with low 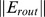 and low 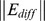. Such networks, the antithesis of star topologies, minimize communication efficiency regardless of the nature of system dynamics and are rarely (if ever) encountered in real-world systems.

The association of certain network architectures with different levels of communication efficiency for routing and diffusion offers a new perspective on network performance. As in our multi-objective optimization experiments, key characteristics of network architecture encountered in real systems may represent the result of selection pressure on efficient communication given the constraints imposed by system dynamics and the cost of building and running the network's infrastructure [38]. Importantly, the dynamics of many real-world systems likely combine aspects of routing and diffusion. For example, depending on context, network communication in social or biological networks can draw on signaling mechanisms for discovering and navigating specific paths, or on mechanisms that aid undirected dispersion or broadcasting. In some systems, diffusion processes can degrade system performance as they allow the propagation of noisy perturbations, while diffusion may be essential in other systems where network elements have little or no knowledge about the global topology. Thus, depending on the balance and significance of routing and diffusion dynamics, network architectures need to achieve a trade-off regarding these two aspects of performance in communication. The results presented here suggest that different levels of specialization for promoting routing or diffusion can be achieved by implementing more distributed and modular versus more centralized and star-like topologies. Modular networks promote routing at the expense of diffusion, by trapping random walkers within local communities while allowing navigation between modules through inter-modular links. Thus, modular networks may be favored when system performance principally relies on routing rather than diffusion. In contrast, star-like topologies, including those containing a rich club of highly interconnected high-degree nodes, may be viewed as a “magnet” for diffusive signal traffic, attracting and dispersing information among more peripheral parts of the network. Star topologies may have a selective advantage when system dynamics utilizes both routing and diffusion. It is important to note that the propensity for real systems to implement modular or star-like topologies is subject to a number of constraints, not explicitly included in the fitness functions we explored, including structural cost, resistance to missing links, density and spatial constraints.

The apparent antagonism between dynamics dominated by diffusion versus routing can be reconciled by introducing the concept of resource efficiency. Even if the elements of a system cannot access global knowledge about network topology, this lack of knowledge can be partly overcome by multiplying the number of particles or messages (resources) used for communication. As more resources are deployed the probability that at least one particle travels along a shortest path increases. Hence, to achieve targeted (and less noisy) communication the addition of resources (involving an expense of material and/or energy) can compensate for a lack of knowledge about network structure. A different way of quantifying the knowledge needed to achieve shortest-path performance utilizes an information-theoretical approach [39]. Here we propose that the amount of resources needed over the entire graph is itself a characteristic descriptor of the graph's architecture. Network topologies not only promote communication by routing or diffusion processes, they can also afford greater resource efficiency, thus helping to reconcile the difference between routing and diffusion. Resource efficiency refers to an aspect of network cost, since resources (messages, signals) tend to consume energy and increase traffic volume. Interestingly, another aspect of network cost that impacts communication efficiency is network density. If adding edges is cheap, but multiplying resources is expensive, dense networks favoring routing over diffusion offer a better compromise between cost and efficiency. If edges are expensive, but increasing resources is more affordable, star or rich-club topologies become the more economical alternative, which more strongly favors systems with diffusive dynamics.

Our approach towards characterizing communication efficiency in networks can be extended in different directions. First, the measures and approaches introduced here could be fully explored for undirected weighted networks. Second, additional measures for characterizing diffusion in complex networks exist, including for instance the entropy rate of a diffusion process [40] and the multiple-passage time [19]. Third, other variants of random walk models can be explored, including those with degree-biased [40], “greedy routing” [41], [42] and intermediate [43] or learning-based [36] routing/diffusion strategies. Finally, all our aggregate measures can be computed for specific node pairs, allowing for instance the identification of specific sets of nodes and communication paths for which the network architecture favors diffusion and/or routing processes.

Future applications and extensions of the framework for characterizing communication efficiency proposed in this article may offer new insights into how complex networks maximize performance when their elements operate with limited knowledge and resources. Such limits are prominently encountered in, for instance, neuronal networks, where the trade-off between cost and efficiency is a major driving force of brain organization [44].

## Supporting Information

Text S1 **Supporting information regarding networks and algorithms.**
(DOCX)

Figure S1
**Pareto fronts from an evolutionary experiment with** 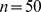 **and** 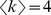 **(a,b) and** 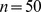 **and** 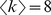 **(c,d).** Pareto front size refers to the number of networks it contains. The age of each member of the Pareto front is defined as the number of consecutive epochs spent in it. The Pareto front age is the average age of each member. (a,c) Evolution of Pareto front sizes for the different fronts. A large pareto front indicates a front expanding across a wide range of the search space, indicating that the in trying to satisfy the multi-objective function evolving networks cannot find a sharp gradient towards improvement. (b,d) Evolution of the Pareto front age for the different fronts. An old Pareto front indicates that the evolutionary process is unable to move the front towards better solutions in order to satisfy the multi-objective function.(TIF)

Figure S2
**Graph evolution driven by network efficiency measures, for networks with** 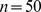 **and** 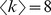 **.** Blue and red squares indicate the reference points of regular lattices and randomized networks, respectively. Green points indicate the initial seed population. Gray circles indicate evolving networks over epochs, with darker shades of gray indicating networks encountered in later epochs. Orange points show Pareto-front (non-dominated) graphs. (a) Snapshots illustrating the expansion of the Pareto fronts at epochs 2, 25, 50, 100, and 200. (b) Final solutions were reached after 1000, 1000, 1000, and 556 epochs for fronts 1, 2, 3, and 4 respectively. Black points denote positions of the example graphs shown in insets. Yellow points show dominated graphs of the final populations. Grey points show coordinates visited by any graph during the evolutionary process at different epochs (denoted by their grey-level).(TIF)

Figure S3
**Graph measures for evolved network topologies with** 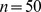 **and** 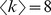 **.** Heat maps are based on a square grid with cells measuring 0.05 units in each dimension. For each cell, graph measures coming from graphs falling on those coordinates at any time point of the evolutionary processes (one for each front) were averaged. (a) Degree entropy. (b) Assortativity. (c) Modularity. (d) Scaled resource efficiency 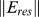.(TIF)

Figure S4
**Graph evolution driven by network efficiency measures, for networks with** 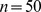 **and** 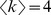 **when degree-sequence is preserved.** Blue and red squares indicate the reference points of regular lattices and randomized networks, respectively. Green points indicate the initial seed population. Orange points show Pareto-front (non-dominated) solutions. Final solutions were reached after 315, 294, 315, and 144 epochs for fronts 1, 2, 3, and 4, respectively. Yellow points show dominated solutions of the final populations. For comparison, evolving networks and Pareto fronts for networks with 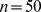 and 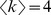 when only density and connectedness are preserved (see Figure 3) are outlined in grey.(TIF)

Table S1 **Graph and normalized efficiency measures for 23 real-world networks including brain, protein interaction, genetic regulatory, social, virtual social, transportation and digital circuit networks.** Compare to Figure 5.
(DOCX)

Movie S1
**A movie showing the complete evolutionary process towards each front for** 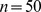 **and** 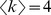 **.** When a front stalls, it means that its corresponding stop criterion has been reached. Blue and red squares indicate the reference points of regular lattices and randomized networks, respectively. Green points indicate the initial seed population. Gray circles indicate evolving networks over epochs, with darker shades of gray indicating networks encountered in later epochs. Orange points show Pareto-front (non-dominated) graphs.(AVI)

Movie S2
**A movie showing the complete evolutionary process towards each front for** 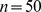 **and** 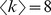 **.** See Movie S1 for details.(AVI)
