# Supplementary material for: Exploring the Morphospace of Communication Efficiency in Complex Networks
Source: PLoS One. 2013 Mar 7;8(3):e58070. doi: 10.1371/journal.pone.0058070 (PMC3591454; doi:10.1371/journal.pone.0058070)
Supplement: Text S1 — Supporting information regarding networks and algorithms. (DOCX) [file pone.0058070.s001.docx]

**Text S1**

**Exploring the Morphospace of Communication Efficiency in Complex Networks**

Joaquín Goñi^a^, Andrea Avena-Koenigsberger^a^, Nieves Velez de Mendizabal^b,c^, Martijn P. van den Heuvel^d^, Richard F. Betzel^a^ and Olaf Sporns^a*^

^a^Department of Psychological and Brain Sciences, Indiana University, Bloomington, Indiana, United States of America.

^b^Department of Medicine, Indiana University, Indianapolis, Indiana, United States of America.

^c^Indiana Clinical and Translational Sciences Institute (CTSI), Indianapolis, Indiana, United States of America.

^d^Department of Psychiatry, University Medical Center Utrecht and Rudolf Magnus Institute of Neuroscience, Utrecht, The Netherlands.

*corresponding author: osporns@indiana.edu

**Construction of Standard Network Models**

**Erdös- Rényi:** Originally, the model stipulates that every pair of nodes is connected with a fixed probability $p$ [1]. Here, given a fixed number of nodes and a desired connectivity density, links are generated with a random-uniform probability. For connected graphs, this is only done in the upper triangular of the adjacency matrix and the matrix is symmetrized afterwards. This model is known to produce connected graphs when $p\geq\frac{\log(n)}{n}$ as $n$ tends to infinity. To keep the density constant, the number of edges was fixed and randomly distributed, instead of fixing the parameter $p$. Connectedness was tested in all the instances produced by this model and only connected graphs were used.

**Barabasi-Albert:** This model is also known as the preferential-attachment model [2]. An initial set of $m_{0}$ fully connected nodes (auto-loops not considered) is created. After that, $iters$ iterations are performed as follows. At each iteration a new node is added to the network. This node will be connected with $m$ existing nodes that will be selected according to their degree. There is a connectivity restriction such that $m_{0}\geq m$. Hence, an existing node $i$ at a given iteration has a probability $k_{i}/\sum_{j} k_{j}$of receiving another connection. This process produces a connected graph of size $n=m_{0}+iters$ and $k\approx2\cdot m$as $n$ tends to infinity [3].

**One dimensional lattice:** A one-dimensional regular lattice is a model that produces connected networks with a uniform degree and high clustering coefficient [4]. After setting the number of nodes $n$ and an even positive integer value to represent $\left\langle k\right\rangle$, the model proceeds as follows. In a clockwise sense, all the nodes are visited. At each visit, the current node is connected to its $\left\langle k\right\rangle/2$ neighbors moving clockwise. This procedure is finished after $n$ iterations. When $\left\langle k\right\rangle$ is even, this model is deterministic.

**Watts-Strogatz:** Rewiring model starting on a regular lattice [4]. It starts with a regular lattice where every node has a fixed degree $k$ $.$ After that, each link is subject to be rewired with a probability$p$. This rewiring procedure is done as follows. A lap of edges of continuous neighbors are evaluated in a clockwise sense. With probability $p$ they are reconnected to a node chosen uniformly at random. If the new link does not yet exist the rewiring is performed, and it is canceled otherwise. This procedure is repeated for more distant neighbors, with $k/2$ giving the number of laps needed. When using low values of $p$, for instance $p\in[0.01,0.1]$, this model produces connected graphs with high clustering coefficient but at the same time short characteristic path length [4]. Parameter $p$ was set to 0.1 in our experiment.

**Real-World Networks**

In this section we provide a brief description of the 23 real networks analyzed and their nature. Major graph theoretical descriptors of each network are shown in Table S1.

**cat_ctx**: Network describing the cortical connectivity among a parcellation of 52 anatomical areas in the cat [5]. Data available at the website <http://www.brain-connectivity-toolbox.net> [6]

**macaque_ctx**: Network describing the cortical connectivity among a parcellation of 71 anatomical areas in the macaque cerebral cortex [7]. Data available at the website <http://www.brain-connectivity-toolbox.net> [6]

**c.elegans**: Neural network of the nematode *Caenorhabditis elegans* as studied in [4]. The network’s nodes represent neurons, and two neurons are considered connected if at least one synapse or gap junction exists between them. Filtering by giant component reduced the network from 306 neurons to 297 neurons. All connections are binary and symmetric.

**human_DSI**: Matrix of inter-regional fiber densities between pairs of anatomical subregions of the human brain, result of averaging over fiber densities for all pairs of ROIs within the regions, and averaging across all five participants. Connection weights are symmetric and were binarized (threshold was set to 0.0001). The dataset was acquired with diffusion spectrum imaging (DSI) and was originally published at [8].

**human_DTI**: Matrix of inter-regional fiber densities between pairs of anatomical subregions of the human brain, result of averaging over fiber densities for all pairs of ROIs within the regions, and averaging across all subjects. Connection weights are symmetric and were binarized (threshold was set to 0.80, i.e. more than 80% of the subjects sharing such connection present). The dataset was acquired with diffusion tensor imaging (DTI). The acquisition and processing steps applied to this dataset are described elsewhere [9].

**yeast_pi** and **a.thaliana_pi**: Giant components of protein interaction networks of yeast and the plant *Arabidopsis thaliana* respectively. Data was downloaded with permission from the website <http://www.soc.duke.edu/~jmoody77/Prot/index.htm>.

**grn_ecoli, grn_mouse, grn_mtuberc, grn_rat** and **grn_human**: Genetic regulatory networks for different species, *Escherichia coli*, *Mus musculus, Mycoplasma tuberculosis*, *Ratus novergicus* and *Homo sapiens*, respectively. The data consists of directed networks where nodes are genes and arcs between two genes captures the interaction of the respective gene product -represented by the source node over the regulatory region of the target gene -target node. Data was published in [10] and is available at <http://info.gersteinlab.org/Hierarchy> .

**dolphins**: A social network of frequent associations (undirected) within a community of 62 dolphins living off Doubtful Sound (New Zealand) [11].

**prison**: In the 1950s John Gagnon collected sociometric choice data from 67 prison inmates [12]. They were asked, "What fellows on the tier are you closest friends with?" They could freely choose as few or as many "friends" as desired. Friendship was converted to an undirected relationship in our study.

**zachary**: Social network of friendships between 34 members of a karate club at a US university in the 1970s [13].

**hiTech**: This network contains the friendship ties among the employees of a small hi-tech computer company [14], which were gathered by means of the following question: “Who do you consider to be a personal friend?” A friendship (edge) was only included in the network if both persons involved acknowledged it. Most friendship nominations happened to be reciprocated. Data is available at Pajek social dataset repository <http://vlado.fmf.uni-lj.si/pub/networks/data/esna> .

**polblogs**: Directed network consisting of hyperlinks between US political weblogs, recorded by Alamic and Glance [15]. These data are available at Mark Newman's website <http://www-personal.umich.edu/~mejn/netdata/netdata/facebook> .

**email**: List of edges of the network of e-mail interchanges between members of the University Rovira i Virgili (Tarragona) [16]. The network can be downloaded at the website <http://deim.urv.cat/~aarenas> . Undirected version of this network emphasizes pairs of users that ever contacted by email, independently of who was the sender and who was de receiver.

**USairport500**: List of edges of the 500 busiest commercial airports in the United States. This dataset was used by Colizza et al. [17]. An edge exists between two airports if a flight was scheduled between them in 2002. Originally the weights correspond to the number of seats available on the scheduled flights and directedness indicated a flight is scheduled from one airport and to another, although a high flight bi-directionality [18] was present. This network is available at the Complex Networks Collaboratory’s website <http://sites.google.com/site/cxnets/data> .

**s344, s641 and s820:** Three directed networks chosen (varying size and density) among 50 electronic sequential logic circuits compiled from ISCAS'89 and ITC'99 sets. These data were used in [19].

**socialNet**: A “Facebook-like” social network from an online community for students at University of California, Irvine [20]. The dataset includes the users that sent or received at least one message. The network is available at Tore Opsahl’s repository <http://toreopsahl.com/datasets>. The version used was named as weighted static one-mode network (weighted by number of messages). It was binarized afterwards to give each edge the identity of contact among two users rather than number of messages.

**Rewiring Methods and Construction of Reference Networks**

Two different populations of reference networks, each matching the size and density of an original network, were created to have lattice-like and random-like reference points in the efficiency space when density and connectedness were preserved. Each ensemble contained 100 graphs. Graphs with low unsigned assortativity, low clustering coefficient and low average path lengths are referred to as highly random-like or disorganized graphs, whereas graphs with high clustering coefficient and high average path lengths are referred to as highly regular 1D lattice-like graphs. Please note that the terms “high” and “low” are relative to limits imposed by the density and/or degree-sequence of the real system under study.

The lattice-like ensemble consisted of creating probabilistic regular lattices matching the average degree $\left\langle k\right\rangle$of the real network. Due to its probabilistic nature, a small density variance was introduced by this method. This method almost preserved connectedness and mostly preserved density whereas produce highly regular graphs.

The random-like ensemble consisted of changing edges in the network in an iterative fashion. At each iteration an existing edge $\{i,j\}$ chosen at random was substituted by a new non existing edge $\{s,t\}$ chosen at random. If the new graph was connected, the change was accepted and reversed otherwise. This process was continued until $5\cdot l$changes were performed successfully with a maximum number of $20\cdot l$ iterations or attempts, $l$ denoting the number of edges in the network. This randomization method preserves density and connectedness whereas produces highly disordered graphs.

When evolving networks while preserving density and connectedness during the multi-objective optimization of the four fronts, as performed to produce Figure 3 and Figure S2, the method described above for the random-like ensemble was used to produce one single change per network at each epoch. When evolving networks while also preserving degree-sequence, as performed to produce Figure S4, the method xswap [21] was used to produce one single change per network at each epoch as follows: two existing edges $\{i,s\}$ and $\{j,t\}$ were chosen at random. If edges $\{i,t\}$ and $\{j,s\}$ did not exist, then edges $\{i,s\}$ and $\{j,t\}$ were removed and edges $\{i,t\}$ and $\{j,s\}$ were created.

**Bibliography**

[1] Erdős P, Rényi A (1960) On the evolution of random graphs. Magyar Tud Akad Mat Kutató Int Közl 5, 17-61.

[2] Barabasi AL, Albert R (1999) Emergence of scaling in random networks. Science 286, 509-512.

[3] Farkas IJ, Derényi I, Barabási AL, Vicsek T (2001) Spectra of "real-world" graphs: beyond the semicircle law. Phys Rev E Stat Nonlin Soft Matter Phys. 64, 026704.

[4] Watts DJ, Strogatz SH (1998) Collective dynamics of 'small-world' networks. Nature 393, 440-442.

[5] Scannell JW, Burns GAPC, Hilgetag CC, O'Neil MA, Young MP (1999) The connectional organization of the corticothalamic system of the cat. Cereb Cortex 9, 277–299.

[6] Rubinov M, Sporns O (2010) Complex network measures of brain connectivity: Uses and interpretations. NeuroImage 52, 1059-1069.

[7] Young MP (1993) The organization of neural systems in the primate cerebral cortex. Proc R Soc Lond B 252, 13–18.

[8] Hagmann P, Cammoun L, Gigandet X, Meuli R, Honey CJ, Wedeen VJ, Sporns O (2008) Mapping the structural core of human cerebral cortex. PLoS Biol. 6, e159

[9] van den Heuvel MP, Sporns O (2011) Rich-club organization of the human connectome. J Neurosci. 2, 15775-86.

[10] Bhardwaj N, Koon-Kiu Y, Gerstein MB (2010) Analysis of diverse regulatory networks in a hierarchical context shows consistent tendencies for collaboration in the middle levels. Proc Natl Acad Sci U S A 107, 6841-6846.

[11] Lusseau D, Schneider K, Boisseau OJ, Haase P, Slooten E, Dawson SM (2003) The bottlenose dolphin community of Doubtful Sound features a large proportion of long-lasting associations, Behavioral Ecology and Sociobiology 54, 396-405.

[12] Macrae D (1960) Direct Factor-Analysis of Sociometric Data. Sociometry 23, 360-371.

[13] Zachary W W (1977) An information flow model for conflict and fission in small groups, J Anthropol Res 33, 452-473.

[14] Krackhardt's D (1999) The ties that torture: Simmelian tie analysis in organizations. Res Sociol Org 16, 183-210.

[15] Adamic LA, Glance N (2005) The political blogosphere and the 2004 US Election. Proceedings of the WWW-2005 Workshop on the Weblogging Ecosystem.

[16] Guimera R, Danon L, Diaz-Guilera A, Giralt F, Arenas A (2003) Self-similar community structure in a network of human interactions. Phys Rev E 68, 065103.

[17] Colizza V, Pastor-Satorras R,Vespignani A (2007) Reaction-diffusion processes and metapopulation models in heterogeneous networks. Nature Phys 3, 276-282.

[18] Barrat A, Barthelemy M, Pastor-Satorras R and Vespignani A (2004) The architecture of complex weighted networks. Proc Natl Acad Sci U S A 101, 3747-3752.

[19] Cancho R F, Janssen C, Sole RV (2001) Topology of technology graphs: small world patterns in electronic circuits. Phys Rev E 64, 046119.

[20] Opsahl T, Panzarasa P (2009) Clustering in weighted networks. Soc Networks 31, 155-163.

[21] Hanhijärvi S, Garriga GC, Puolamäki K (2009) Randomization Techniques for Graphs. Proc of the 9th SIAM Int Conf on Data Mining (SDM '09), 780-791.
