# Supplementary figures and images for: Exploring the Morphospace of Communication Efficiency in Complex Networks
Source: PLoS One. 2013 Mar 7;8(3):e58070. doi: 10.1371/journal.pone.0058070 (PMC3591454; doi:10.1371/journal.pone.0058070)

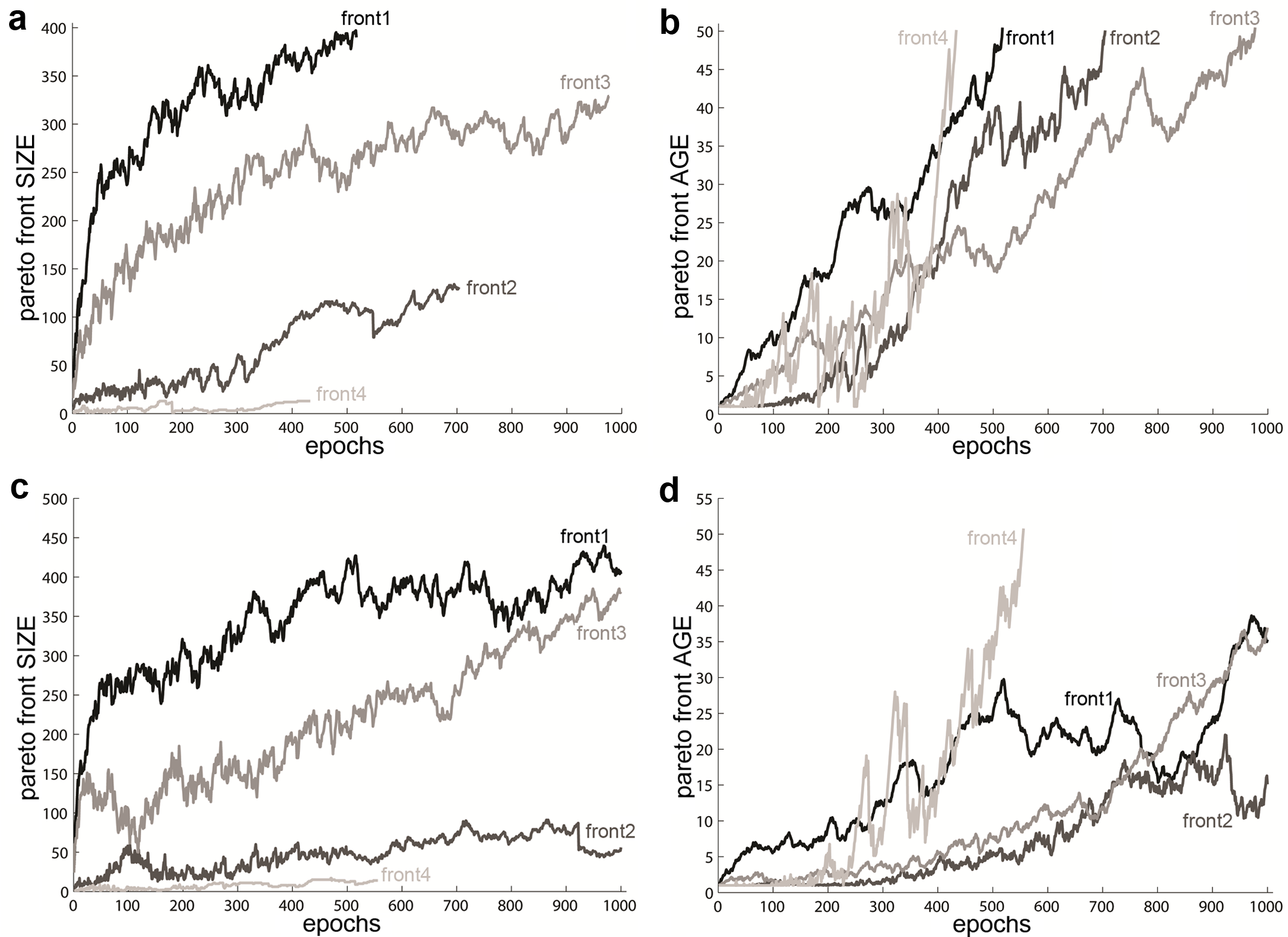

Supplement: Figure S1 — Pareto fronts from an evolutionary experiment with and (a,b) and and (c,d). Pareto front size refers to the number of networks it contains. The age of each member of the Pareto front is defined as the number of consecutive epochs spent in it. The Pareto front age is the average age of each member. (a,c) Evolution of Pareto front sizes for the different fronts. A large pareto front indicates a front expanding across a wide range of the search space, indicating that the in trying to satisfy the multi-objective function evolving networks cannot find a sharp gradient towards improvement. (b,d) Evolution of the Pareto front age for the different fronts. An old Pareto front indicates that the evolutionary process is unable to move the front towards better solutions in order to satisfy the multi-objective function. (TIF) [file pone.0058070.s002.tif]

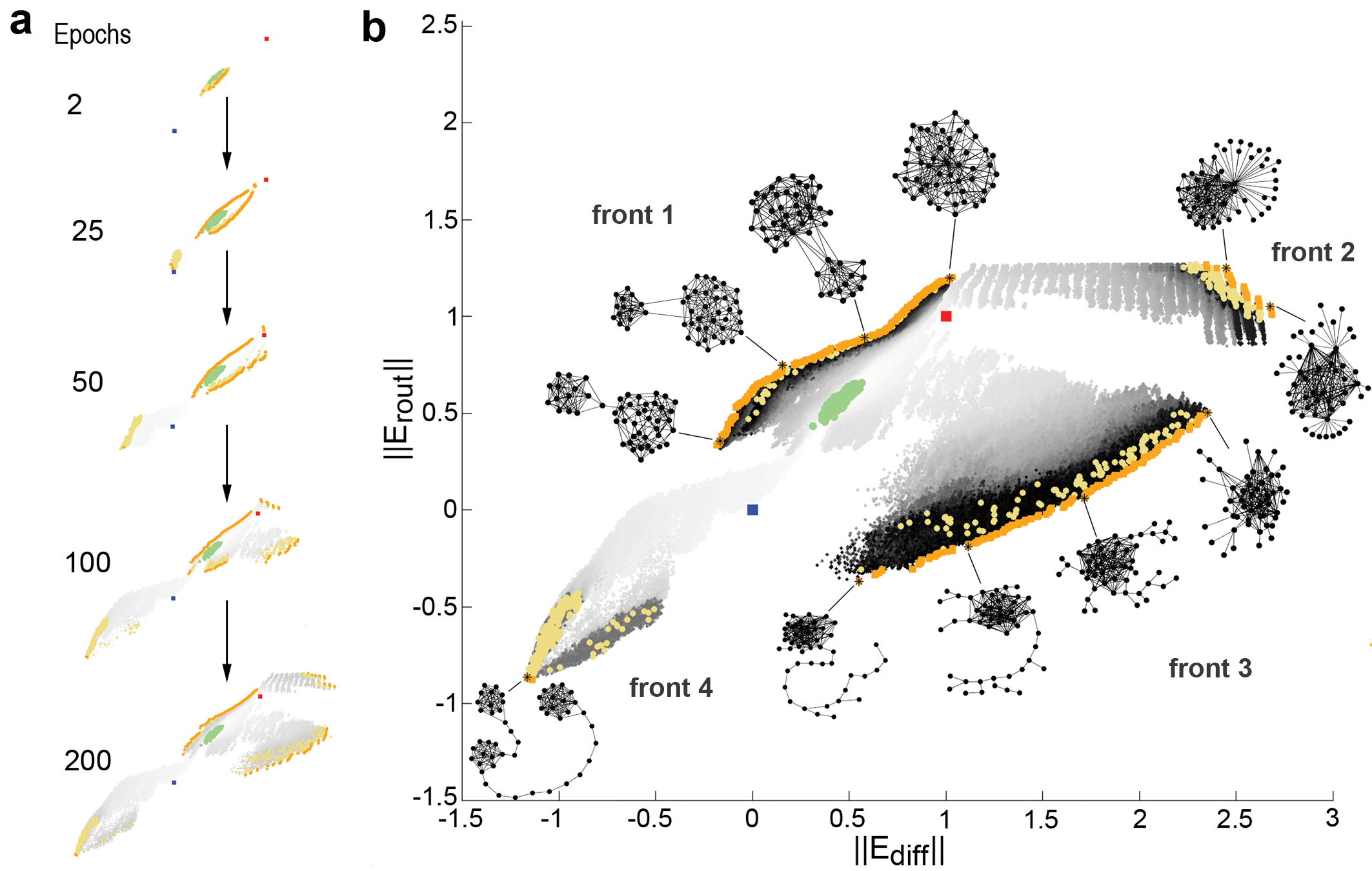

Supplement: Figure S2 — Graph evolution driven by network efficiency measures, for networks with and . Blue and red squares indicate the reference points of regular lattices and randomized networks, respectively. Green points indicate the initial seed population. Gray circles indicate evolving networks over epochs, with darker shades of gray indicating networks encountered in later epochs. Orange points show Pareto-front (non-dominated) graphs. (a) Snapshots illustrating the expansion of the Pareto fronts at epochs 2, 25, 50, 100, and 200. (b) Final solutions were reached after 1000, 1000, 1000, and 556 epochs for fronts 1, 2, 3, and 4 respectively. Black points denote positions of the example graphs shown in insets. Yellow points show dominated graphs of the final populations. Grey points show coordinates visited by any graph during the evolutionary process at different epochs (denoted by their grey-level). (TIF) [file pone.0058070.s003.tif]

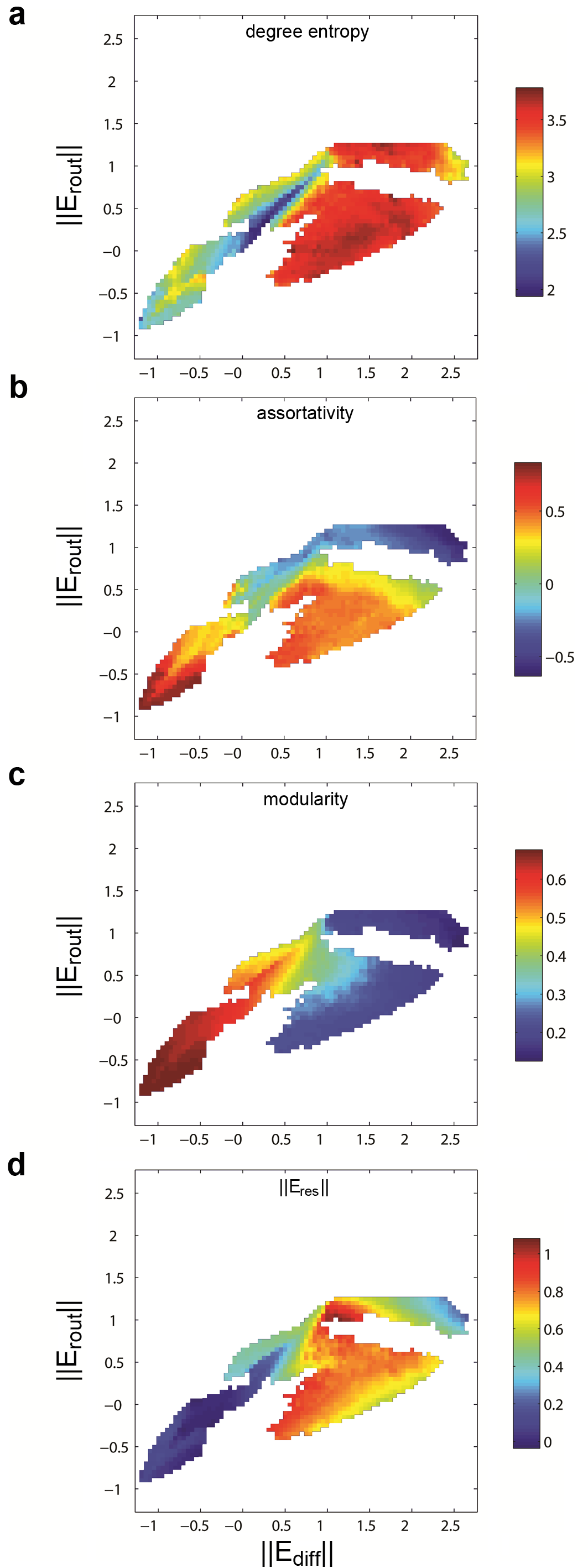

Supplement: Figure S3 — Graph measures for evolved network topologies with and . Heat maps are based on a square grid with cells measuring 0.05 units in each dimension. For each cell, graph measures coming from graphs falling on those coordinates at any time point of the evolutionary processes (one for each front) were averaged. (a) Degree entropy. (b) Assortativity. (c) Modularity. (d) Scaled resource efficiency . (TIF) [file pone.0058070.s004.tif]

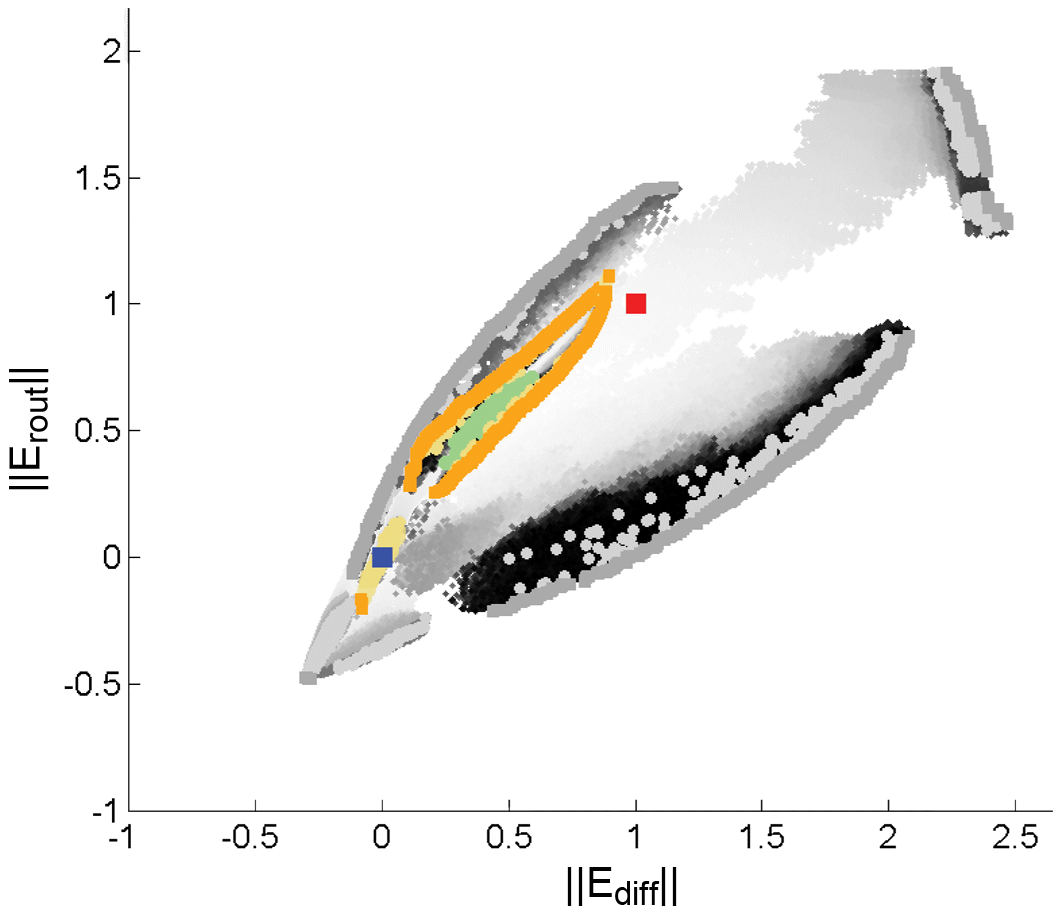

Supplement: Figure S4 — Graph evolution driven by network efficiency measures, for networks with and when degree-sequence is preserved. Blue and red squares indicate the reference points of regular lattices and randomized networks, respectively. Green points indicate the initial seed population. Orange points show Pareto-front (non-dominated) solutions. Final solutions were reached after 315, 294, 315, and 144 epochs for fronts 1, 2, 3, and 4, respectively. Yellow points show dominated solutions of the final populations. For comparison, evolving networks and Pareto fronts for networks with and when only density and connectedness are preserved (see Figure 3) are outlined in grey. (TIF) [file pone.0058070.s005.tif]
