# Supplementary material for: Exploring the Morphospace of Communication Efficiency in Complex Networks
Source: PLoS One. 2013 Mar 7;8(3):e58070. doi: 10.1371/journal.pone.0058070 (PMC3591454; doi:10.1371/journal.pone.0058070)
Supplement: Table S1 — Graph and normalized efficiency measures for 23 real-world networks including brain, protein interaction, genetic regulatory, social, virtual social, transportation and digital circuit networks. Compare to Figure 5. (DOCX) [file pone.0058070.s006.docx]

| Category | Network | $n$ | $\left\langle k\right\rangle$ | $\left\langle spl\right\rangle$ | $\left\Vert E_{rout}\right\Vert$ | $\left\Vert E_{diff}\right\Vert$ |
| --- | --- | --- | --- | --- | --- | --- |
|  | cat_ctx | 52 | 19.80 | 1.63 | 0.89 | 0.83 |
|  | macaque | 71 | 12.33 | 2.24 | 0.68 | 0.88 |
| brain | c.elegans | 297 | 14.46 | 2.46 | 1.00 | 1.06 |
|  | human_DSI | 66 | 8.46 | 2.65 | 0.61 | 0.68 |
|  | human_DTI | 66 | 8.18 | 2.70 | 0.64 | 0.69 |
| protein | yeast pi | 1458 | 2.67 | 6.81 | 1.25 | 1.40 |
| interaction | a.thaliana pi | 515 | 3.65 | 4.67 | 1.15 | 1.28 |
|  | grn_ecoli | 1373 | 4.30 | 3.71 | 1.51 | 1.45 |
| genetic | grn_mouse | 1185 | 4.03 | 3.93 | 1.40 | 1.56 |
| regulatory | grn_mtuberc | 687 | 2.40 | 4.65 | 2.14 | 1.82 |
|  | grn_rat | 524 | 4.12 | 3.63 | 1.35 | 1.56 |
|  | grn_human | 3101 | 4.41 | 3.38 | 1.73 | 1.72 |
|  | dolphins | 62 | 5.12 | 3.35 | 0.70 | 0.96 |
| social | prison | 67 | 4.23 | 3.35 | 0.84 | 0.83 |
|  | zachary | 34 | 4.59 | 2.41 | 1.05 | 1.03 |
|  | hiTech | 33 | 5.52 | 2.36 | 0.80 | 1.33 |
|  | polblogs | 1222 | 27.35 | 2.73 | 0.92 | 1.16 |
| virtual social | socialNet | 1893 | 14.61 | 3.05 | 1.04 | 1.26 |
|  | email | 1133 | 9.62 | 3.60 | 0.95 | 1.13 |
| transportation | USairport500 | 500 | 11.92 | 2.99 | 0.95 | 1.22 |
|  | s344 | 184 | 3.09 | 5.46 | 0.86 | 0.61 |
| digital circuits | s641 | 427 | 2.59 | 9.32 | 0.77 | 0.74 |
|  | s820 | 312 | 4.88 | 3.48 | 1.14 | 0.96 |

**Table S1**. Graph and normalized efficiency measures for 23 real-world networks including brain, protein interaction, genetic regulatory, social, virtual social, transportation and digital circuit networks. Compare to Figure 5.
